# Key thermally dimorphic fungal pathogens: shaping host immunity

**DOI:** 10.1098/rsob.210219

**Published:** 2022-03-09

**Authors:** Maxine A. Höft, Lucian Duvenage, J. Claire Hoving

**Affiliations:** ^1^ CMM AFRICA Medical Mycology Research Unit, Institute of Infectious Diseases and Molecular Medicine (IDM), University of Cape Town, Cape Town 7925, South Africa; ^2^ Department of Pathology, Faculty of Health Sciences, University of Cape Town, Cape Town 7925, South Africa; ^3^ MRC Centre for Medical Mycology at the University of Exeter, Geoffrey Pope Building, Stocker Road, Exeter EX4 4QD, UK

**Keywords:** antifungal immunity, dimorphic fungi, C-type lectin receptor, C‐type lectin receptors (CLR), toll‐like receptors (TLR)

## Abstract

Exposure to fungal pathogens from the environment is inevitable and with the number of at-risk populations increasing, the prevalence of invasive fungal infection is on the rise. An interesting group of fungal organisms known as thermally dimorphic fungi predominantly infects immunocompromised individuals. These potential pathogens are intriguing in that they survive in the environment in one form, mycelial phase, but when entering the host, they are triggered by the change in temperature to switch to a new pathogenic form. Considering the growing prevalence of infection and the need for improved diagnostic and treatment approaches, studies identifying key components of fungal recognition and the innate immune response to these pathogens will significantly contribute to our understanding of disease progression. This review focuses on key endemic dimorphic fungal pathogens that significantly contribute to disease, including *Histoplasma*, *Coccidioides* and *Talaromyces* species. We briefly describe their prevalence, route of infection and clinical presentation. Importantly, we have reviewed the major fungal cell wall components of these dimorphic fungi, the host pattern recognition receptors responsible for recognition and important innate immune responses supporting adaptive immunity and fungal clearance or the failure thereof.

## Graphical abstract

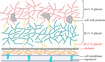

## 1. Introduction

Fungi can be found in almost every environment on earth and are particularly abundant in organic substrates such as soil and plant debris. Despite the millions of fungal species that humans are exposed to, only an estimated 600 species can colonize our bodies or cause opportunistic infection [1]. Infection may arise from commensal overgrowth leading to fungal dysbiosis or from exposure to environmental fungal pathogens [2], with the increasing number of immunocompromised patients being particularly vulnerable. Over millions of years, fungi have evolved and adapted to survive stressors in their environment. They have developed mechanisms to alter their cell shape and form, as well as stress responses and developmental strategies as they react to triggers in their immediate environment. Although many pathogenic fungi are able to alter their morphology as part of their lifecycle, relatively fewer species are considered to be truly dimorphic. The morphological shift in these pathogens is triggered by a change in temperature when entering the host. These pathogens are thermally dimorphic fungi.

These dimorphic fungi have adapted to switch between multicellular filamentous growth or hyphae to unicellular growth forms or yeasts. Dimorphic fungi are found in three main phyla, namely: Ascomycota, Basidiomycota and Zygomycota. There are different environmental stimuli that trigger this strict transition to generate either a hyphal or yeast morphology. While there are many fungi that show aspects of dimorphism, in this review, we will focus on a specific group of fungi belonging to the phyla Ascomycota that exhibit a trait known as thermal dimorphism. Thermally dimorphic fungi are generally found in the soil growing at 22–25°C as mycelia that generate conidia, which are released into the air by wind and soil disruptions along with hyphal fragments. Transition into the yeast phase (or spherules for *Coccidioides* spp.) occurs at 37°C upon inhalation by the host into the lungs, which can lead to infection [3]. The symptoms of infection can vary; they can be mild and undetected or develop into more serious conditions such as pneumonia, acute respiratory distress syndrome and disseminated disease [3]. The severity of disease depends on exposure and the immune status of the individual, with immunocompromised patients at higher risk of severe disease and death. Thermally dimorphic fungi produce conidia that are responsible for geographical dispersal and host infection. However, it is the transition into yeast form that drives pathogenicity, as these organisms have evolved to alter their cell wall components and proteins to survive at mammalian body temperature and evade immune responses. The main thermally dimorphic pathogens of humans are globally distributed and include *Histoplasma capsulatum, Blastomyces dermatiditis, Coccidioides immitis/posadasii, Paracoccidioides brasiliensis/lutzii, Talaromyces marneffei* (formerly known as *Penicillium marneffei*), *Sporothrix schenckii* and newly identified, *Emergomyces* spp. These organisms are primary pathogens, but for this review, we will focus on key pathogens that cause the most infections, including, *Histoplasma Coccidioides* and *Talaromyces* spp.

## 2. Prevalence and route of infection

Very few fungal infections are notifiable diseases and therefore, precise information on their prevalence throughout the world is very limited. In fact, until recently, Coccidioides was the only nationally notifiable disease with the CDC in the USA. However, the WHO is generating a priority list of fungal pathogens of public health importance, and this will likely improve the epidemiological data generated for these pathogens. Histoplasmosis, caused by *Histoplasma capsulatum,* is primarily a respiratory infection, but in patients with impaired T-cell function, it can progress to a life-threatening systemic infection. In some parts of Latin America, the deaths resulting from histoplasmosis among HIV/AIDS patients outnumber those from tuberculosis [4]. The distribution of *H. capsulatum* is worldwide but it is highly endemic in central North America (especially the USA Midwest) and South America. Considering the high burden of both tuberculosis and advanced HIV disease in sub-Saharan Africa, it is possible that the incidence of Histoplasmosis is higher than previously anticipated, but not being detected. Coccidioidomycosis, also known as Valley fever, is a disease caused by *Coccidioides immitis* (endemic to Northern Mexico and both central and southern California) and *Coccidioides posadasii* (detected throughout Arizona, Mexico, Texas and other regions of South America). Exposure to the fungus is common, with an estimated 40% of the population in hyperendemic areas infected [5]. Most immunocompetent individuals exposed are asymptomatic, some experiencing mild symptoms, and they can clear the infection without medical intervention. However, in certain endemic areas, *Coccidioides* spp. has been reported to be a common cause of community-acquired pneumonia, requiring antifungal treatment [6]. Valley fever, referring to the infection of the lungs, is more common, but in severe cases, disseminated coccidioidomycosis can occur. While *Histoplasma* spp. and *Coccidioides* spp. can cause disease in both immunocompetent and immunocompromised individuals, *Talaromyces marneffei* [7] is an AIDS-defining illness in South and Southeast Asia. The endemic regions of the fungal disease include Northern Thailand, Southern China, Vietnam, Northern India, Hong Kong and Taiwan [8]. Like *Histoplasma* spp., *T. marneffei* can disseminate from the lung to other organs, with the potential to reactivate at a later stage, but infection in healthy individuals is not common.

*Histoplasma* spp. are soil-dwelling fungi, found particularly in moist soils of river valleys and in bird or bat guano, with particularly high concentrations in caves [9]. Similarly, *Coccidioides* spp. are found in dust, but the low numbers isolated from soil suggest that this organism is better adapted to an animal host. It has not yet been established if *Talaromyces* spp. occur in the soil, but it is known to infect wild rodents such as the bamboo rat, constituting a reservoir in endemic countries [10,11]. Infection with *Histoplasma* spp. and *Talaromyces* spp. occurs through the inhalation of conidia and a shift in temperature inside the host triggers a switch to the pathogenic yeast form. Uniquely, *Coccidioides* spp. form arthoconidia, which transition into pathogenic spherules in the host [12].

Most patients infected with *Histoplasma* spp. have no symptoms. In cases of inhalation of a large inoculum of conidia, acute infection may develop, characterized by fevers, malaise, dry cough and lymphadenopathy. Systemic infection develops in approximately 1 in 2000 acute cases and clinical presentation can be quite diverse due to the ability of the fungus to colonize several organs: lung, bone marrow, skin and gastrointestinal tract [13]. For C*occidioides* spp., infected individuals generally present with symptoms 1–3 weeks after exposure, which may include the development of a cough, shortness of breath, fever, night sweats, fatigue, headache, muscle or joint pain and skin rash on legs or upper body. Common infected tissues include bones, joints, meninges and skin; dissemination to pericardium, abdomen, adnexa and larynx has been reported, although less commonly [14]. Similarly, *Talaromyces* spp. are usually cleared by the immunocompetent host within 2–3 weeks. However, impaired T cell function leads to systemic infection, as shown in T cell-deficient hosts [15]. Systemic infection with *Talaromyces* spp. is life-threatening, with one of the highest mortality rates of AIDS-defining illnesses [16]. Clinical manifestations of disseminated infection include fever, weight loss, skin lesions and hepatomegaly. Skin lesions on the face and neck are characteristic (approximately 85% of patients) as the fungus colonizes the skin [17]. Misdiagnoses are common in the absence of widely available diagnostic tools [18] and highlight the important need to improve tests, which should be widely available.

## 3. The dimorphic fungal cell wall

**Figure 1.**
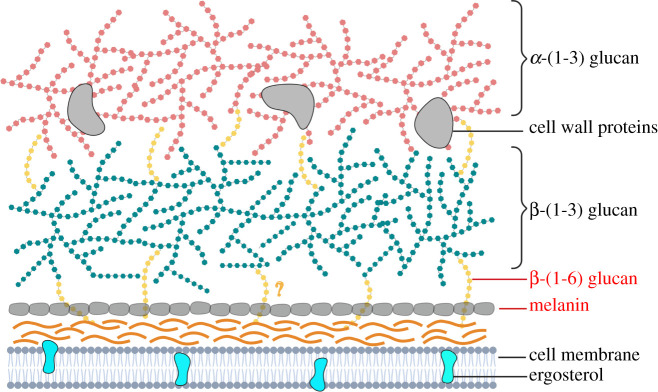
Schematic representation of the cell wall of select dimorphic fungi. All common components are shown here, though not all may be present in each species. Chitin and β-(1,3) glucan form the structural core of the cell wall, which may be cross-linked by β-(1,6) glucan. The type of melanin and its distribution can vary by species and its position shown here is speculated, as is the location of β-(1,6) glucan. Alpha-(1,3) glucan is common on the cell wall exterior of dimorphic fungi. Created with BioRender.com.

The fungal cell wall is a protective barrier against environmental stresses and functions to maintain intracellular turgor pressure. Across fungal species, there are carbohydrate polymers in common that serve to maintain the structural integrity of the cell wall. Thermally dimorphic fungi share many of these core components such as chitin—a polymer of N-acetyl glucosamine—and β-(1,3) glucan (table 1). β-(1,6) glucan, shown in *Saccharomyces cerevisiae* to be lower in abundance and to cross-link multiple cell wall layers, such as chitin to β-(1,3) glucan layers (table 1) [36]. These polymers form a structural inner layer, upon which an outer cell wall layer can be formed (figure 1). Specific to many dimorphic fungi, α-(1,3) glucan is present in this outer cell wall layer [37–39]. While mannans are common in other fungal organisms, they have not yet been described for dimorphic fungi. For *Aspergillus fumigatus*, galactomannan is present as a linear α-mannan which is modified with short chains of β-(1,5) glucofuranose residues [40]. While galactomannan detection in urine is the basis of an enzyme immunoassay diagnostic test for Histoplasmosis [41], whether this is a component of the dimorphic cell wall or only present in a soluble form needs to be clarified. Melanin is also present in the dimorphic organisms discussed here [42,43]. Within dimorphic fungal species, there can be considerable variation in the cell wall composition of the different morphological forms (mycelia, conidia and yeast). The filamentous forms have adapted to survival as free-living environmental organisms, whereas the yeast forms survive at elevated temperatures within the mammalian host, interacting with the immune system [44]. Components of the cell wall which are recognized as foreign by pathogen recognition receptors (PRRs) of the host immune system are referred to as pathogen-associated molecular patterns (PAMPs).

**Table 1.** Major pathogen-associated molecular patterns (PAMPs) found on the cell walls of thermally dimorphic fungal pathogens and associated pathogen recognition receptors (PRRs). Relevant references are indicated in the text. Abbreviations: Hsp60, heat-shock protein 60; Yps3, yeast phase-specific protein-3; CR3, complement receptor 3; VLA-5, very late antigen-5; TLR, toll-like receptor; Mp1, mannoprotein 1; DC-SIGN, dendritic cell-specific ICAM-3-grabbing non-integrin; Gp70, glycoprotein-70 kDa; BAD1, blastomyces adhesin-1; MCL, murine macrophage C-type lectin; MR, mannose receptor; SOWgp, spherule outer‐wall glycoprotein.

pathogen	phase	PAMP	PRR	references
*Histoplasma capsulatum*	yeast	[β-(1,3) glucan]?	Dectin-1	[19]
		Hsp60	CR3	[20]
		cyclophilin-A	VLA-5 (dendritic cells)	[21]
		Yps3	TLR2	[22]
		yeast DNA	TLR7, TLR9 (dendritic cells)	[23]
*Talaromyces marneffei*	conidia	unknown	TLR1, TLR2, TLR4, TLR6	[24]
		Mp1 mannoprotein	unknown	[25]
	conidia/yeast	unknown	CR3	[26]
	yeast	Unknown	DC-SIGN (dendritic cells)	[27]
		N-acetyl-β-D-glucosaminyl groups	unknown	[28]
*Coccidioides* spp.	spherule	unknown	TLR2	[29]
		[β-(1,3) glucan]?	Dectin-1	[30]
		mannose	MR	[31,32]
		SOWgp	?	[33–35]

## 4. Pathogen-associated molecular patterns

### 4.1. Chitin

Chitin is a widely conserved structural polymer in the fungal cell wall. For *T. marneffei*, chitin has been shown to be an essential fungal cell wall component. Cánovas and colleagues demonstrated the importance of myosin (*MyoB*) in chitin deposition. Defects in *MyoB* were associated with chitin defects leading to the absence of conidiophore cell types [45]. Although conserved, chitin content can vary greatly between species and between chemotypes of the same species. Furthermore, the level of chitin in the cell wall also changes upon dimorphic switching. For *Coccidioides posadasii*, particular subsets of chitin synthases are responsible for the production of chitin at different stages of differentiation. Comparing the expression of seven synthase genes, Mandel *et al.* identified the pattern of expression during morphogenesis. While the genes *CpCHS2*, *CpCHS3* and *CpCHS6* were expressed during the saprobic phase, *CpCHS1* and *CpCHS4* were associated with the pathogenic phase. *CpCHS5* and *CpCHS7* were found in both phases [46]. The host environment can also influence the chitin cell wall content, as shown recently for *Histoplasma* spp. [47]. Here Assunção *et al.* demonstrate that low zinc availability in macrophages increases the chitin and glycan content in fungal cell wall. This causes a smoother cell surface and is suggested to increase pathogenicity by inhibiting the production of cytokines released by the host. Despite the importance of these findings, and even though several proteins in the human host have been shown to interact with chitin, the role of chitin as a PAMP in dimorphic fungal infections remains unclear.

### 4.2. Melanin

Melanins are a diverse group of high-molecular dark brown or black pigments that act as fungal armour. The two most commonly found melanins in fungi are eumelanin DOPA-melanin and allomelanin-derived DHN-melanin. Little is known about the organization of melanin in cell walls of dimorphic pathogens [48], but the presence of melanin has raised interest in whether it is associated with fungal virulence. For example, *Sporothrix schenckii* expresses melanin in both the conidial and yeast phase during *in vivo* infection [49]. Like *S. schenckii*, pigmented *H. capsulatum* conidia and yeast have tufts on their exterior surface and resemble the granules seen on *S. schenckii* conidia [50]. The *in vitro* production of melanin by *H. capsulatum* conidia without the addition of phenolic precursors suggests that the pigment may be DHN melanin [42]. However, the genome of *T. marneffei*, which encodes several laccases associated with both DOPA- and DHN-melanin synthesis, suggests that both are types of melanin are synthesized [51–53]. Melanin may also confer resistance to antifungal drugs*.* Several fungi are stimulated to produce melanin *in vitro* when grown in the presence of L-DOPA. *H. capsulatum* was shown to increase resistance to Amphotericin B and caspofungin if stimulated to generate melanin under these conditions [54].

### 4.3. Beta-glucans

β-glucans are the most abundant fungal cell wall polysaccharides. Due to the highly immunogenic nature of β-(1,3) glucan, concealment or ‘masking’ is a desirable immune evasion strategy, as demonstrated with *H. capsulatum* [55]. By contrast, β-(1,6) glucan has relatively low abundance in the cell wall and is not considered to have a significant role in the immune response. However, in other non-dimorphic fungal organisms, β-(1,6) glucan is incorporated in branch points in β-(1,3) glucan chains and can influence the degree of branching and molecular weight of β-glucan polymers. Therefore, β-(1,6) glucan is thought to be an important factor in the immuno-stimulatory activity of β-(1,3) glucan, but this has not been shown for thermally dimorphic fungi [56,57].

### 4.4. Cell wall proteins

Proteins anchored to the cell wall have important functions in cell wall maintenance, nutrient acquisition and stress resistance. Some of these may be recognized by the host immune system and trigger phagocytosis, which, in the case of some fungal pathogens, is a survival strategy due to their preference to multiply within macrophages, and aids in dissemination in the host. Heat-shock proteins (Hsps) are an example of cell wall proteins recognized by the immune system. For example, Hsp60 of *H. capsulatum* is recognized by complement receptor 3 on macrophages and neutrophils, triggering phagocytosis [20]. Cell wall proteins, such as Mp1 in *T. marneffei*, are often highly immunogenic and species-specific and are therefore attractive candidates for vaccine and diagnostics development [58]. Similarly, in *Coccidioides* spp., a spherule-abundant protein (Pmp1), secreted fungal aspartyl proteases (Pep1) and recombinant β-1,3-glucanosyltransferase (Gel1) have all shown promise as effective vaccine targets in the murine model of coccidioidomycosis [59–61]. Therefore, identifying cell wall proteins in vaccine strategies is incredibly important.

### 4.5. Alpha glucans

Increasing evidence suggests that most pathogenic dimorphic fungi display α-(1,3) glucan on their cell wall surface [38,39,55]. However, more information is needed to determine the specific detail of α-(1,3) glucan's contribution to the cell wall structure of other dimorphic organisms such as *Coccidioides* spp. Furthermore, very little information is available about α-glucan recognition by the host and the associated immune response. However, this cell wall polymer has been shown to be required for virulence in *Histoplasma* spp. [55,62,63]. Removal of α-(1,3) glucan from the outer cell wall increases immune recognition by Dectin-1, suggesting that α-(1,3) glucan may mask underlying β-(1,3) glucan [64]. Furthermore, the β-glucanase Eng1 trims exposed β-(1,3) glucan and therefore blocks recognition by the host receptor Dectin-1 [19]. Considering that α-(1,3) glucan is a major cell wall component in dimorphic fungi, this is an important area for future research.

## 5. Dimorphic fungal recognition and associated host response

**Figure 2.**
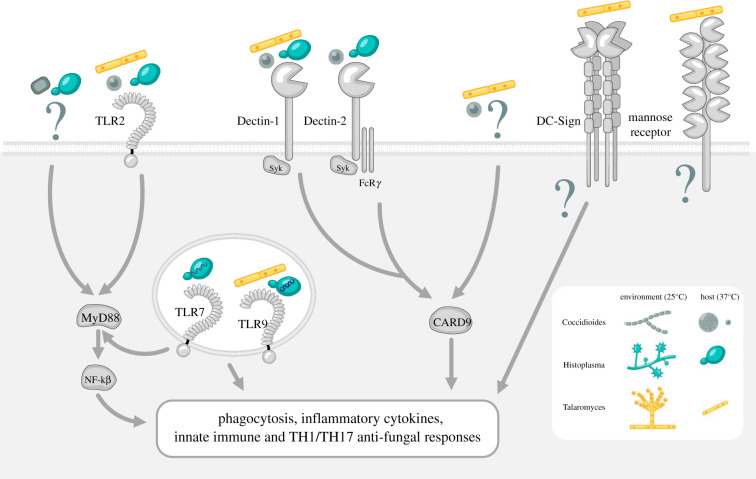
Innate recognition of select thermally dimorphic fungal pathogens and the downstream activation pathways. PRRs on innate immune cells recognize PAMPs during fungal infection. The main PRRs involved in dimorphic fungal recognition are TLRs (such as TLR2, TLR7 and TLR9) and CLRs (such as Dectin-1, Dectin-2, DC-sign and MR). Upon binding and dimorphic fungal recognition of specific fungal PAMPs by TLRs and CLRs, certain downstream intracellular signalling pathways are induced resulting in multiple antifungal immune responses. CARD9, Caspase recruitment domain‐containing protein 9; MyD88, Myeloid differentiation primary response 88; NF‐κB, nuclear factor kappa. Created with BioRender.com.

Phagocytes widely express membrane-bound PRRs that can directly recognize PAMPs on cell wall components of fungi. The nature of fungal dimorphism presents a challenge for immune detection and activation because the form in which the organism enters the host changes. As described above, *Histoplasma, Coccidioides* and *Talaromyces* spp. all express chitins and α- and β-glucans in their outer cell wall. It is postulated that these cell components are recognized by a variety of host toll-like receptors (TLRs) and C-type lectin receptors (CLRs) to elicit strong inflammatory responses from local immune cells (figure 2). However, receptors for chitin, α-1,3-glucan or galactose polymers remain to be identified. Several TLRs have been shown to recognize fungal PAMPs and play a role in antifungal immunity, triggering inflammatory responses, mainly working together with CLRs (e.g. Dectin-1 and mannose receptor (MR)).

An elegant review by Ray & Rappleye [65] highlights *Histoplasma* spp. recognition by host cells and can likely be applied to other similar dimorphic pathogens. However, so much remains unknown, particularly the signalling pathways and cell types involved in the recognition of and response to these dimorphic pathogens. The ability of the innate immune system to trigger an adaptive T cell response is critical to resolution of infection. However, equally important is the role of innate receptors. MyD88, an adaptor protein vital to multiple TLR signalling pathways, was critically required for the host response to *Histoplasma* spp. MyD88-deficient mice were unable to control the fungal burden and were more susceptible than wild-type, with reduced early inflammatory cytokine production. Interestingly, cell-specific deletion of MyD88 from alveolar macrophages or dendritic cells did not affect mouse survival, suggesting an additional cell type or compensatory mechanisms for clearance [66]. TLR2 binds to a protein, Yps3, expressed on the surface of the yeast phase of *H. capsulatum* [22]. Yps3 stimulated nuclear factor kappa B (NF-κB) production via TLR2 in both HEK293T cells and in murine microglia; however, further research is required using lung phagocytes or *in vivo* models to determine the precise role of TLR2. A recent study showed that dendritic cells were able to mount a type I interferon response via TLR7 and TLR9 and proved to be a major driver of the T cell activation required to clear *Histoplasma* spp. infection [23].

A role for TLR recognition was also demonstrated using myeloid differentiation primary response 88 (MyD88^−/−^) mice for *Coccidioides* spp. infection. MyD88^−/−^ mice were more susceptible to *Coccidioides* spp. infection, with a higher fungal burden in the lung and spleen and impaired cytokine production [67]. Here, interleukin-1R1 (IL-1R1) also uses MyD88 in signalling but not TLR2 was required for clearance. While peritoneal macrophages elicited from TLR2-deficient mice had impaired cytokine production in response to *Coccidioides* spp. spherules [68], TLR2^−/−^ mice were able to control infection with no increase in susceptibility. Similarly, the susceptibility of mice lacking TLR4 or IL-18R was not affected [69]. By contrast, IL-1R1-deficient mice were reported to have increased disseminated fungal burden, suggesting signalling via IL-1R1 and Myd88 may play a role in coccidioidomycosis immunity in mice [70]. *Talaromyces marneffei* conidia are recognized by several PRRs. A study using monoclonal antibodies against PRRs on human monocytes to investigate binding to *T. marneffei* conidia showed that the MR, TLR1, TLR2, TLR4, TLR6, CD14, CD11b and CD18 were all involved in phagocytosis [24]. Recently, a study in AIDS patients in China linked talaromycosis severity with single-nucleotide polymorphisms in TLR2 but not in TLR3 or TLR9 [71].

CLRs have been shown to play key roles in the recognition of fungal pathogens. While evidence is limited for dimorphic fungi, studies suggest a role for CLRs in mediating immunity. CLRs recognize PAMPs on fungal cell wall components and initiate downstream signalling pathways that regulate innate immune responses such as phagocytosis, respiratory burst (resulting in reactive oxygen species (ROS) production), inflammasome activation, neutrophil extracellular trap formation (NETosis), antigen presentation and dendritic cell maturation, as well as the production of inflammatory mediators (e.g. cytokines, chemokines, eicosanoids, etc.) [72]. Both Dectin-1 and Dectin-2 were shown to interact with *H. capsulatum,* although recognition by Dectin-1 was limited by β-glucan masking*.* Both receptors, especially Dectin-2, were found to be important for NOD‐, LRR‐ and pyrin domain‐containing protein 3 (NLRP3) inflammasome activation in dendritic cells, leading to IL-1β production. While Dectin-1, MR, DC-SIGN/specific intercellular adhesion molecule‐3‐grabbing nonintegrin‐related 1 (SIGNR1) and Fc*γ*R were shown not to be involved in the phagocytosis of yeasts by murine macrophages [73]. Dectin-1 maintained the ability to mediate the production of TNF-α and IL-6 by associating with the complement receptor type 3 (CR3) in murine macrophages [74]. It has long been known that phagocytosis of *H. capsulatum* by macrophages is dependent on recognition by CR3 and not β-glucan recognition [75]. However, it was only later shown that the major ligand facilitating this binding was heat-shock protein 60 (Hsp60) on the yeast cell wall [20]. For dendritic cells, however, phagocytosis is not mediated through CR3 but rather by the fibronectin receptor VLA-5 [76], which recognizes cyclophilin A, a protein on the surface of the yeast cell wall [21].

Studies investigating the role of CLRs have suggested a role in controlling coccidioidomycosis. First, *in vitro* studies using RAW 264.7 macrophages overexpressing Dectin-1 infected with *C. posadasii* spherules produced an increased cytokine response. Similarly, using antibodies to block Dectin-1 in elicited mouse peritoneal macrophages impaired proinflammatory cytokine production [68]. Bronchoalveolar lavage fluid collected from Dectin-1-deficient mice after *Coccidioides* spp. infection contained reduced levels of IFN-γ and IL-17a cytokines [29]. Associated with this reduction was an increase in fungal burden in the lung and spleen of Dectin-1^−/−^ mice. Linked to the role of Dectin-1, caspase recruitment domain‐containing protein 9 (CARD9) was shown to be vital in triggering Th-1- and Th-17-mediated immune responses towards coccidioidomycosis. Mice that lack CARD9 were highly susceptible to pulmonary and subcutaneous *Coccidioides* spp. infection and failed to produce protective immunity to the disease [70]. By contrast, Dectin-2 and MR had no apparent role in the resistance to *Coccidioides* spp. infection in mice, as there was no difference in susceptibility to infection in mice lacking either or both receptors [30]. However, *in vitro* studies using MR/Dectin-1^−/−^ peritoneal macrophages and bone marrow‐derived dendritic cells (BMDCs), less proinflammatory cytokine production was seen in response to infection with spherules [30]. Furthermore, *in vitro* studies using human dendritic cells show that MR recognizes *C. posadasii* spherules and initiates cytokine production [31,32].

For *Talaromyces spp.,* a very recent study has suggested a role for Dectin-1 in initiating signalling through the activation of Syk, which triggered phosphorylation of IκBα and NF-κB. This study was carried out *in vitro* using THP-1 macrophages and heat-killed *T. marneffei* [77]. The fact that heat-killed *T. marneffei* was used and that there is the potential for unnatural exposure of β-glucan polysaccharides to Dectin-1 should be considered. Therefore, further evidence using *in vivo* models is required to draw conclusions. It was found that MR was important for yeast phagocytosis in human monocyte-derived macrophages, and that DC-SIGN was involved in adhesion to dendritic cells [27]. Interestingly, another study found that MR was not involved in the binding of heat-killed yeasts in murine macrophages. Perhaps heat killing the organism affects the ligand recognized by MR. Phagocytosis was strongly inhibited by competition with wheatgerm agglutinin, suggesting that the yeast phase is recognized by exposed N-acetyl-β-D-glucosaminyl groups [28]. These studies showed that cell wall differences between the morphological forms of dimorphic fungi led to differential engagement of host PRRs. Lastly, the integrin CR3 (a heterodimer of CD11b and CD18) has been shown to recognize a wide variety of fungal pathogens. In response to *T. marneffei*, murine macrophages were shown to upregulate the expression of CD11b*,* and the inhibition of CD11b significantly reduced phagocytosis of the yeast. This recognition led to the secretion of IFN-*γ*, TNF-α, IL-4, IL-10 and IL-12 [25,26]. The surface ligand in *T. marneffei* that binds to CR3 has not yet been identified.

Receptors on phagocytic cells such as macrophages, neutrophils, monocytes and dendritic cells play a vital role in activating immune cells and promoting fungal killing. *Histoplasma* and *Talaromyces* spp. are both intracellular pathogens engulfed by host cells. Although *Coccidioides* spp. has an intracellular component during infection, the interaction with host cells is predominantly extracellular. Alveolar macrophages are among the first innate immune cells with which *H. capsulatum* comes into contact in the lung. Recent evidence suggests that dendritic cells are the major antigen-presenting cell during *H. capsulatum* infection and are important for initiating T-helper type 1 (Th1) responses required to clear the organism [78]*.* Taken up by macrophages and neutrophils, *H. capsulatum* yeasts proliferate intracellularly, and within these cells may disseminate to other organs via blood or the lymphatic system. Innate immune cells that are not activated are ineffective in killing intracellular yeasts; only once a Th1 cell-mediated adaptive response has developed may phagocytes contain the infection [79]. Even then, infection may persist and remain dormant in granulomas and can be reactivated following compromised immunity such as immunosuppressive therapy or HIV/AIDS [80]. After the inhalation of the airborne fungal particles, the host phagocytic cells engulf *Coccidioides* spp. arthroconidia. The arthroconidia is triggered to transition into spherule initials and eventually into multinucleate spherules [81]. Endospores are formed within the spherules that become enlarged, causing the cell wall to rupture upon maturation. Endospores are then released to infect nearby tissue capable of forming new spherules, repeating the life cycle [82]. Phagocytes are able to ingest arthroconidia, sphere initials and endospores; however, mature spherules are too large to engulf. Therefore, *Coccidioides* spp. have both intracellular and extracellular relationships with the host. Endospores and sphere initials are more susceptible to killing and inhibition of growth by activated phagocytes [83,84]. Lastly, Dong *et al.* have established that cytokines produced by innate immune cells are critical for resolution of *T. marneffei* infection in AIDS patients [85]. In a study of 41 AIDS patients infected with *T. marneffei*, cytokine profiles were tracked over a six-month period after initiation of antifungal therapy. Inflammatory cytokines TNF-α, IFN-γ, IL-6, IL-12, IL-18 and IL-1β were important for resistance of the disease. However, excessive inflammatory responses led to poor patient outcomes. *T. marneffei* proliferates within macrophages to evade host immunity. A recent study using a zebrafish embryo model found that the conidia are predominantly taken up by macrophages, which supports growth in the yeast phase and protects against the myeloperoxidase fungicidal activity of neutrophils [86]. However, caution should be taken when interpreting these data as the zebrafish model would not represent a host temperature of 37°C and therefore not completely represent true yeast form. Furthermore, *T. marneffei* was recently shown to promote M2-like polarization of human macrophages, thereby promoting fungal survival. By downregulating SOCS3 expression, or degrading SOCS3, the organism was able to suppress host protective M1 activation. Here, the authors showed that by inhibiting TLR9 activation, this response was partially blocked. This study suggests that the antifungal ability of macrophages depends on their activation status [87].

## 6. Virulence-associated traits of dimorphic fungi

As β-(1,3) glucan is a key PAMP for fungal recognition and clearance, many fungal pathogens conceal this PAMP to avoid an immune response [55,88]. *H. capsulatum* has several mechanisms to avoid a Dectin-1 response. A layer of α-(1,3) glucan covers underlying β-(1,3) glucan [89], and the yeast may also secrete endoglucanases such as Eng1 to trim back any exposed β-(1,3) glucan [19]. Independent studies of *H. capsulatum* chemotype II strains, in which α-(1,3) glucan production has been disrupted, have been shown to exhibit reduced virulence in mouse models of infection [62,90]. A forward genetic screen identified Hsp82 as another important virulence factor, highlighting the importance of heat-shock proteins in resistance to stresses imposed by the host [90]. To survive the ROS produced in the phagosome of macrophages, *H. capsulatum* produces superoxide dismutase (SOD) and catalase [91]. Without Sod3, an extracellular SOD produced during infection, *H. capsulatum* cannot survive in activated macrophages. Nearly all mice infected with a lethal dose of wild-type yeast survive for approximately 5 days, whereas almost all mice survive infection with the same dose of the *sod3*Δ strain after two weeks [92]. The virulence mechanisms of *Coccidioides* spp. are largely unknown; however, arthroconidia significantly increase in size when transitioning into spherules, which contain between 100 and 300 endospores. The large size of the spherule makes phagocytosis by innate cells challenging. The spherule also contains three unique genes that contribute to virulence factors and host tissue damage: *BLG2, SOWgp* and *MEP1* [33–35]*.* BLG2 cleaves β-1,3-glucan from the spherule cell wall to allow for expansion and growth. Spherule outer-wall glycoprotein (SOWgp) enables the spherule to adhere to host cells by binding to laminin, fibronectin and collagen in the extracellular matrix. The host immune system can recognize SOWgp; however, a Th2 response is activated that assists pathogen survival. Endospores are coated with SOWgp during spherule maturation; however, MEP1 degrades SOWgp, which is another mechanism of preventing immune recognition [3]. For *Talaromyces* spp., a distinguishing feature when grown *in vitro* is the production of a soluble red pigment. Laccases are responsible for the formation of melanin-like pigments. The deletion of a laccase gene *pbrB* in *T. marneffei* resulted in a strain that was more readily phagocytosed by THP-1 human monocyte cells and stimulated increased cytokine production, suggesting that laccases may have a function in immune evasion [93]. Furthermore, the cell wall mannoprotein Mp1 was found to be important for virulence. Mice infected with *Mp1* knockout mutants all survived, in contrast to the 100% mortality when infected with the wild-type strain [94]. To conclusively demonstrate that this was associated with increased survival within macrophages, a direct comparison of phagocytosis rates of wild-type and mutant fungal strains would need to be made. A recent study also found that Mp1 was able to sequester proinflammatory lipid arachidonic acid, thus interfering with host signalling. Mp1 is abundant on the surface of conidia, highly antigenic and found in the sera of infected patients. An enzyme-linked immunosorbent assay with high sensitivity and specificity using an anti-Mp1 antibody was developed, demonstrating that mannoproteins could be an attractive target for diagnostic assay development for other fungi [58].

## 7. Conclusion, open questions and perspectives

Despite the increase in prevalence and high mortality rates of invasive dimorphic fungal infections, they continue to be misdiagnosed or underreported. Medical mycology has been a neglected research area in general, but even within this, endemic dimorphs receive far less attention than other fungi such as *Candida* or *Cryptococcus.* Due to climate change and increased human movement, the endemic range of these pathogens is expanding, and the number of infections may continue to increase [95,96].

The importance of understanding how our immune system interacts with these dimorphic fungal pathogens will provide critical insight into potential vaccine development and therapeutic interventions. Innate immunity is particularly important, as it prevents the vast majority of exposures to fungi in the environment from developing into systemic disease. When systemic disease does occur, innate immune responses shape the induction of adaptive immunity, which is required to clear infections. We have described host–pathogen interactions for some of the most common thermal dimorphic fungal pathogens. However, this field of research is relatively new, and many questions remain unanswered. Much information thus far has been gained from *in vitro* studies using cell lines*,* while *in vivo* data using animal models or clinical studies are lacking. Any observed interactions of fungi with cell monocultures *in vitro* may not necessarily influence disease progression in a meaningful way; nevertheless, we include this data to serve a record for future studies striving to elucidate the significance of these interactions using *in vivo* models (table 1).

TLR signalling is a common response to dimorphic fungi, although the role in triggering an immune response requires further investigation. Recognition of *H. capsulatum* yeast DNA by TLR7/TLR9 is a fascinating recent discovery [23], highlighting the adaptation of the immune system to the intracellular lifestyle of many of these pathogens, which could be exploited in future therapeutic strategies. Recognition by CR3 is common to *Histoplasma, Talaromyces* and *Blastomyces* spp. Recognition of β-(1,3) glucan by Dectin-1 does not seem to have a major role in phagocytosis of dimorphic fungi, but rather, may have a role in initiating the adaptive response and clearance once these ligands become more exposed. In general, the role of CLRs is not as clear for these pathogens, as for other medically important fungi. The cell wall of most dimorphic fungi contains α-(1,3)-glucan, which has been shown to mask β-(1,3) glucan recognition; however, receptors for this ligand have not yet been identified, and the manner in which this cell wall component directly shapes the immune response should be a priority for investigation, given its widespread occurrence. As listed in table 1, several fungal ligands of innate immune receptors remain uncharacterized. Some components of the cell wall of these pathogens, which induce a response, engage as yet unidentified receptors. An understanding of these interactions is necessary for the development of potential vaccines. Early diagnostic tools are a cost-effective strategy for preventing severe disease in at-risk populations. Development of diagnostic kits requires an understanding of immunogenic fungal components and their receptors, whether these are innate immune receptors or antibodies. The development of inexpensive and point-of-care diagnostics is particularly important in low- to middle-income countries, where the resources and expertise for PCR identification may not be widely available. Despite these considerable challenges and unanswered questions, recent improvements in diagnostics and their increased availability in endemic areas are promising signs. For example, the development of the enzyme-linked immunosorbent assay for diagnosis of disseminated Histoplasmosis and it simplementation in Latin America are considerable steps forward in recognizing and fighting infection [97], together with creating awareness of the problem, such as the global call for talaromycosis to be recognized as a neglected tropical disease [98]. With commitment from funders, policy makers, researchers and industry, the control of these endemic dimorphic pathogens is feasible and thereby protecting vulnerable populations.

## Data Availability

This article has no additional data.
